# Milk Formula Enriched with Sodium Butyrate Influences Small Intestine Contractility in Neonatal Pigs

**DOI:** 10.3390/nu14204301

**Published:** 2022-10-14

**Authors:** Monika Słupecka-Ziemilska, Stefan Grzegorz Pierzynowski, Paulina Szczurek, Kateryna Pierzynowska, Piotr Wychowański, Blanka Seklecka, Maciej Koperski, Anna Starzyńska, Dominika Szkopek, Janine Donaldson, Krzysztof Andrzejewski, Jarosław Woliński

**Affiliations:** 1 Department of Human Epigenetics, Mossakowski Medical Research Institute Polish Academy of Sciences, 02-106 Warszawa, Poland; 2 Department of Medical Biology, Institute of Rural Health, 20-090 Lublin, Poland; 3 SGP + Group, 231 32 Trelleborg, Sweden; 4 Department of Biology, Lund University, Sölvegatan 35, 223 62 Lund, Sweden; 5 Department of Animal Nutrition and Feed Sciences, National Research Institute of Animal Production, 32-083 Balice, Poland; 6 Department of Animal Physiology, The Kielanowski Institute of Animal Physiology and Nutrition, 05-110 Jabłonna, Poland; 7 Division of Oral Surgery and Implantology, Department of Head and Neck, Institute of Clinical Dentistry, Oral Surgery and Implantology Unit, Fondazione Policlinico Universitario A. Gemelli IRCCS-Universita Cattolica del Sacro Coure, 00168 Rome, Italy; 8 Adamed Pharma S.A., 05-152 Pieńków, Poland; 9 Departament of Oral Surgery, Medical University of Gdańsk, 7 Dębinki Street, 80-211 Gdańsk, Poland; 10 Large Animal Models Laboratory, The Kielanowski Institute of Animal Physiology and Nutrition, 05-110 Jabłonna, Poland; 11 School of Physiology, Faculty of Health Sciences, University of the Witwatersrand, 7 York Road, Parktown, Johannesburg 2193, South Africa; 12 Department of Orthopedics and Traumatology, Veteran’s Memorial Hospital, Medical University of Łódź, 90-549 Łódź, Poland

**Keywords:** sodium butyrate, intestinal contractility, neonates, pigs, cholinergic signaling

## Abstract

Butyrate, a by-product of gut bacteria fermentation as well as the digestion of fat in mother’s milk, exerts a wide spectrum of beneficial effects in the gastrointestinal tissues. The present study aimed to determine the effects of sodium butyrate on small intestine contractility in neonatal piglets. Piglets were fed milk formula alone (group C) or milk formula supplemented with sodium butyrate (group B). After a 7-day treatment period, isometric recordings of whole-thickness segments of the duodenum and middle jejunum were obtained by electric field stimulation under the influence of increasing doses of Ach (acetylocholine) in the presence of TTX (tetrodotoxin) and atropine. Moreover, structural properties of the intestinal wall were assessed, together with the expression of cholinergic and muscarinic receptors (M1 and M2). In both intestinal segments (duodenum and middle jejunum), EFS (electric field stimulation) impulses resulted in increased contractility and amplitude of contractions in group B compared to group C. Additionally, exposure to dietary butyrate led to a significant increase in tunica muscularis thickness in the duodenum, while mitotic and apoptotic indices were increased in the middle jejunum. The expression of M1 and M2 receptors in the middle jejunum was significantly higher after butyrate treatment. The results indicate increased cholinergic signaling and small intestinal growth and renewal in response to feeding with milk formula enriched with sodium butyrate in neonatal piglets.

## 1. Introduction

In mammals, the gastrointestinal tract (GIT) is both structurally and functionally immature at birth; therefore, its development continues in the early postnatal period. During this time, the intestine undergoes adaptive processes associated with significant changes in structural growth and remodeling of the intestinal epithelium as well as functional maturation, including changes in intestinal absorption and motor activity. It should also be mentioned that approximately one-third of preterm and low birth weight infants display delayed maturation of motility patterns within the gut [1].

Breastfeeding is the best nutritional strategy for infants. In contrast to milk formula, mother’s milk provides the newborn with biologically active compounds, which optimize gut microbial colonization, support immune maturation and gastrointestinal growth and development, program metabolism, and influence cognitive development. Moreover, feeding with mother’s milk prevents both short-term and long-term health complications, including necrotizing enterocolitis in preterm neonates as well as obesity, diabetes, and cardiovascular diseases later in adult life. However, there is still a relatively large group of infants who require milk formula feeding, and thus efforts should be made to improve its nutritional quality [2].

Butyrate is a four-carbon, short-chain fatty acid (SCFA) that originates from gut bacterial fermentation. During the suckling period, the majority of butyrate is formed as a by-product from the gastric digestion of fat in the mother’s milk [3]. Similar to other short-chain fatty acids, butyrate is not only a breast milk metabolite but is also present in breast milk itself and is part of milk fat globules, the digestion of which begins in the stomach [4,5,6]. Undoubtedly, the fermentation of milk oligosaccharides represents another source of butyrate [7]. However, the fecal butyrate content of breast-fed infants is much lower compared to that of formula-fed babies [8,9].

Butyrate exerts a number of beneficial effects on the intestinal epithelium that extend far beyond the colon. Butyrate serves as a fuel source for epithelial cells, exerts trophic effects on the intestinal mucosa, reduces gut inflammation, strengthens the gut barrier function, stimulates secretion of pancreatic and jejunal enzymes, modifies ileal microbiota, and facilitates apoptosis of colonic cancer cells [10,11,12]. Moreover, butyrate has been shown to influence gut motility. In studies on adult rats, supplementation with butyrate increased colonic muscle contraction in both in vitro and ex vivo studies [13]. Similar results were obtained in canine [14] and feline colonic smooth muscles in vitro [15]. On the other hand, reduced contractile activity was reported in the rat colonic musculature after ingestion of butyrylated starch [16] as well as in the stomach of pigs following infusion of butyric acid into the ileum [17]. Studies in rat pups showed that butyrate enemas enhanced colonic motility by modulating the phenotype of the myenteric network [18,19]. Unfortunately, there is lack of information on the effect of orally administrated butyrate on duodenal and jejunal motility in newborn mammals. Nevertheless, previous studies have shown that administration of butyrate has a positive effect on the remodeling of small intestine mucosa in newborn piglets fed milk formula [20]. Moreover, supplementation of total parenteral nutrition with butyrate led to improved structural and functional adaptation of the jejunum in a pig model of short bowel syndrome [21].

These data highlight the important role of butyrate in the maintenance of intestinal health and at the same time indicate the lack of evidence on how butyrate affects GI motility in neonates. Thus, the aim of this study was to determine the effects of sodium butyrate on the motor activity of duodenum and jejunum in newborn piglets fed milk formula.

## 2. Materials and Methods

### 2.1. Animals and Experimental Design

The study was approved by the University of Lund Ethics Review Committee on Animal Experiments (Approval # M169-14). Animal husbandry and care were performed in accordance with the recommendations in the Guide for the Care and Use of Laboratory Animals of the National Institutes of Health.

The experiment used 16 newborn male piglets (Yorkshire × Swedish Landrace) × Hampshire) obtained from a SPF local herd (Vindfälle 810, 268 68 Röstånga, Sweden). They were born on time and without complications, with an average body weight (BW) of 1.95 ± 0.22 kg. For the first 24 h of life, the piglets were kept with their sows and then transported to the Lund University animal facility and housed in individual cages. The cages were equipped with an artificial sow system (Mamina 4 Special, Mazzolari Impiantistica, Casteldidone, Italy), which provided each piglet with fresh milk formula (Lakti R, Trouw Nutrition, Nutreco, Eindhoven, NL) every 75 min (20 times/day). The amount of milk formula administered was calculated based on daily BW gain and protein requirements of newborn piglets (11.0–11.3 g/kg).

After an adjustment period of 24 h, piglets were randomly allocated to two groups (*n* = 8): control group (C), which was fed milk formula alone, and the experimental group (B), which was fed milk formula and supplemented with sodium butyrate (Merck, Schuchardt, Hohenbrunn, Germany) at a dose of 0.3 g/100 g dry matter. Piglets’ BW was recorded daily. At the end of a 7-day treatment period, piglets were euthanized by an overdose of pentobarbiturate (Nembutal, Abbott Scandinavia AB, Solna, Sweden). The GIT tissues were removed and collected for later analysis.

### 2.2. Sample Preparation

Samples of the duodenum and middle jejunum were immediately fixed either in Bouin solution for morphometric analysis or in 10% buffered formalin for immunohistochemistry for 24 h. The 1–2 cm segments were routinely embedded in paraffin, sectioned (4.5 μm), and placed on silane-treated glass slides. For immunohistochemistry and TUNEL assay, antigen retrieval was performed by microwave-heated incubation (20 min, 500 W) in citrate buffer (pH 6.0).

### 2.3. Intestinal Contractility In Vitro

Duodenum and middle jejunum sections (15 mm long) were collected and immediately placed in ice-cold Krebs–Henseleit solution. The intestinal segments were mounted in organ baths (25 mL) (Letica Scientific Instruments, Hospitalet, Spain) filled with Krebs–Henseleit solution, maintained at 37 °C, and aerated with carbogen (95% O_2_, 5% CO_2_) [18]. The intestinal strips were connected to isotonic transducers under a load of 0.75 g, which were coupled to a data acquisition system (PowerLab/4e, ADInstruments, Bella Vista, Australia) and computer software (Chart v4.01, ADInstruments, Bella Vista, Australia). After an equilibration period of 1 h, with intermittent washing every 15 min to recover spontaneous activity, the segments were treated with 10^−5^ M acetylocholine (ACh, Sigma-Aldrich, St. Louis, MO, USA) for 1 min. Next, the tissues were washed and allowed to equilibrate and then treated with cumulative doses of Ach (10^−9^–10^−4^ M) for dose–response contractility recordings (Figure S1). Half of the randomly selected tissues were pretreated with atropine (ATR, 10^−5^ M), (Sigma-Aldrich, St. Louis, MO, USA).

Neuronal-mediated contractile effect was evoked through electric field stimulation (EFS). Following a 1 h equilibration period, tissues underwent EFS (EXP-ST-01, Experimetria, Budapest, Hungary) for 10 s at 90 V with different frequencies (0.5, 5, and 50 Hz), with 1 min intervals between each pulse (Figure S1). Half of the intestinal segments were pretreated with tetrodotoxin (TTX, 10^−5^ M) (Abcam, Cambridge, UK). At the end of each experiment, the samples were treated with 10^−5^ M ACh to check the viability of the tissue and then with 10^−4^ M isoproterenol to control tissue relaxation.

### 2.4. Intestinal Morphology

Duodenum and middle jejunum sections were fixed as described above, immersed in xylene and descending grades of alcohol, and routinely stained with H&E. Three slides were randomly selected for each intestinal section, and 30 measurements of the muscularis layer thickness were performed using a light microscope coupled with a digital camera and image analysis software (Lucia Software v.4.60, Laboratory Imaging, Praha, Czech Republic).

### 2.5. TUNEL Assay

Intestinal epithelium apoptosis was assessed in paraffin-embedded middle jejunum segments using the TUNEL method as was described in detail in the manufacturer’s instructions (ApopTag, Chemicon, Rolling Meadows, IL, USA). Apoptotic cells were identified and counted in at least 15 high-power fields using a confocal microscope (LSM Pascal, Zeiss, Germany, 400× magnification). The apoptotic index was assessed as the ratio of apoptotic cells to the total number of epithelial cells of villi or crypts, respectively. The nuclei of cells were stained with DAPI.

### 2.6. Immunohistochemistry

Middle jejunum segments were incubated with primary polyclonal rabbit anti-Ki67 antibodies (Abcam, Cambridge, UK, 1:50) for 30 min at room temperature in a humid chamber [22]. After washing with phosphate-buffered saline (PBS), the slides were incubated with secondary antibodies conjugated with horseradish peroxidase enzyme. The slides were washed in PBS and stained with 3,3′-diaminobenzidine and then washed in distilled water and counterstained with hematoxylin. The staining procedure was performed using the EnVision+ system (DakoCytomation, Glostrup, Denmark). Crypt cells undergoing cell division were expressed as mitotic index and calculated as a percentage of Ki67-positive cells among all epithelial cells of the crypt cross section counterstained with hematoxylin and recognized in 15 high-power fields under a light microscope (Axioskop 40, Zeiss, Jena, Germany) at a magnification of 400×.

### 2.7. Western Blotting

Immunostaining of cholinergic muscarinic receptors 1, 2, and 3 (M1, M2, and M3, respectively) was performed in the middle jejunum segments frozen immediately after collection at −80 °C [23]. Prior to analysis, the samples were thawed and harvested in lysis buffer supplemented with protease and phosphatase inhibitor cocktails (Sigma-Aldrich, Darmstadt, Germany). The samples were held at 4 °C for 30 min for complete cell dissociation and centrifuged at 14,000 rpm for 30 min. Supernatants were aspirated, and protein quantification was performed using a Bio-Rad protein assay dye reagent (Bio-Rad Laboratories Inc., Hercules, CA, USA). Protein samples (50 μg) were fractionated by sodium dodecyl sulfate polyacrylamide gel electrophoresis and transferred to polyvinylidene difluoride membranes (Sigma-Aldrich, Germany). After membrane blocking in Tris-buffered saline (TBS) containing 0.5% Tween 20 with 5% nonfat dry milk, the membranes were incubated overnight at 4 °C with primary antibodies against M1, M2, and M3 (1:250) (Abcam, Cambridge, UK,) and β-actin as a “housekeeping” protein (1:10,000) (Santa Cruz Biotechnology, Inc., USA). Next, the membranes were washed three times in TBS for 5 min per wash and incubated with fluorophores-conjugated secondary antibodies, IRDye 680CW, and IRDye 800CW for M1, M2, and M3, respectively (1:5000). This was followed by three washes in TBS for 5 min per wash. The proteins recognized by the antibodies were detected with an Odyssey Infrared Imaging System (LI-COR Biosciences, Lincoln, NE, USA) with a scan resolution of 169 lm and the intensity set at 4. Quantitative integrated optical density analysis was performed.

### 2.8. Statistical Analysis

The data were expressed as mean ± standard deviation (SD) with significance defined as *p* < 0.05. Mann–Whitney test or unpaired *t*-test was used to indicate statistical differences between groups. All analyses were performed using GraphPad Prism version 7.0a (GraphPad Software Inc., San Diego, CA, USA).

## 3. Results

### 3.1. Piglet Growth and Intestinal Morphometry

**Figure 1:**
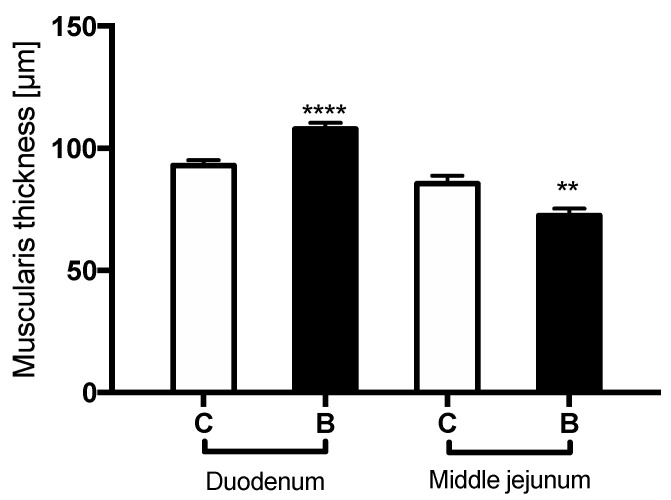
The thickness of the muscularis mucosa muscle layer (μm) in the duodenum and middle jejunum of control (C) and sodium-butyrate-treated piglets (B). Values are given as means ± SD; * indicates statistical differences between groups. In the duodenum, sodium butyrate led to a significant increase in the thickness of the muscle layer (**** *p* < 0.0001). On the contrary, a significant reduction in the muscularis thickness was observed in the middle jejunum after sodium butyrate administration (** *p* = 0.0023).

The initial BW of piglets was 1.92 ± 0.25 and 1.99 ± 0.18 kg in group C and B, respectively. Within the 7-day treatment period, a significant increase in BW of the piglets was noted in both groups, with an average daily gain of 81 ± 59 and 159 ± 55 g in group C and B, respectively (*p* = 0.0031). The final BW of piglets was 2.50 ± 0.38 and 3.10 ± 0.32 kg in group C and B, respectively (*p* = 0.001). The supplementation of milk formula with sodium butyrate had no effect on milk intake. The results of the thickness of the muscularis mucosa layer have been show on the Figure 1.

### 3.2. Spontaneus Intestinal Contractility

**Figure 2:**
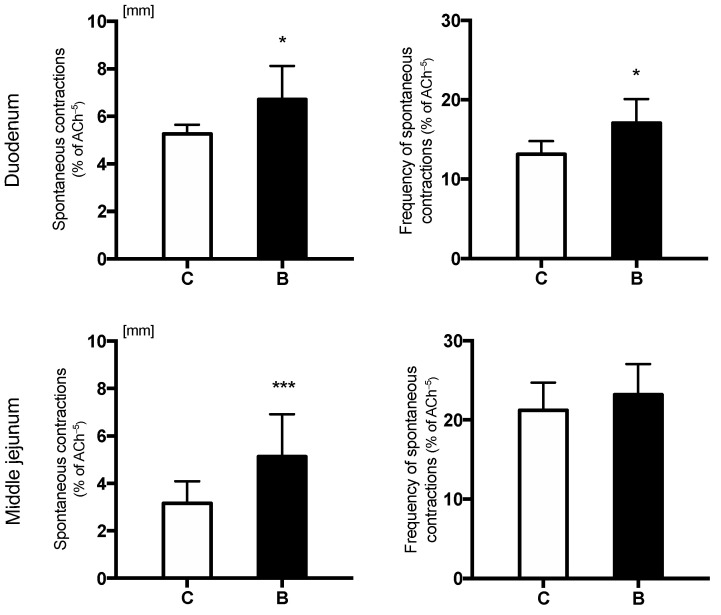
Amplitude of contractions (mm) of isolated duodenal and middle jejunum fragments in control (C) and sodium-butyrate-treated piglets (B). Values are given as means ± SD; * indicates statistical differences between groups when * *p* < 0.05, *** *p* < 0.001.

The oral administration of sodium butyrate significantly increased the amplitude of spontaneous contractions in both duodenal and middle jejunum segments (Figure 2). The frequency of spontaneous contractions was significantly higher in group B compared to group C in only the duodenum (Figure 2).

### 3.3. Electric Field Stimulation

In the duodenal and middle jejunum segments, EFS impulses resulted in a frequency-dependent increase in the amplitude of contractions (Table 1, Figure S2). Additionally, in both the intestinal segments studied, significantly increased amplitude of contractions were observed in butyrate-treated piglets compared to control piglets following all three EFS frequencies. Preincubation with TTX decreased amplitude of contractions compared to intestinal segments that were not pretreated. However, when comparing the amplitude of contractions between the two groups following TTX pretreatment, the amplitude was significantly increased in sodium-butyrate-treated piglets (B) compared to the control group (C) in both the duodenum and middle jejunum (Table 1).

### 3.4. ACh-Stimulated Intestinal Contractility

Increasing cumulative doses of ACh (from 10^−9^ to 10^−4^ M) caused a dose-dependent increase in contractile amplitude of whole-thickness intestinal samples of duodenum and middle jejunum in both groups of piglets (Table 2, Figure S1). In the control group, the effective stimulation of the duodenal and jejunum segments started from the addition of ACh at a dose of 10^−5^ M. In the duodenal segments from the group treated with sodium butyrate, the lowest effective dose of ACh was 10^−6^ M, while the effective dose of ACh was 10 times lower in the jejunum (10^−7^ M). The contractile response to cumulative doses of ACh was significantly higher in the butyrate-treated group compared to the control group in both the duodenum and middle jejunum. Moreover, pretreatment with atropine led to a significant decrease in the responsiveness in both intestinal segments from both experimental groups. The responsiveness after blockage with atropine was also significantly higher in the duodenum of group B compared to that of the control piglets (Table 2).

### 3.5. The Mitotic and Apoptotic Indices

**Figure 3:**
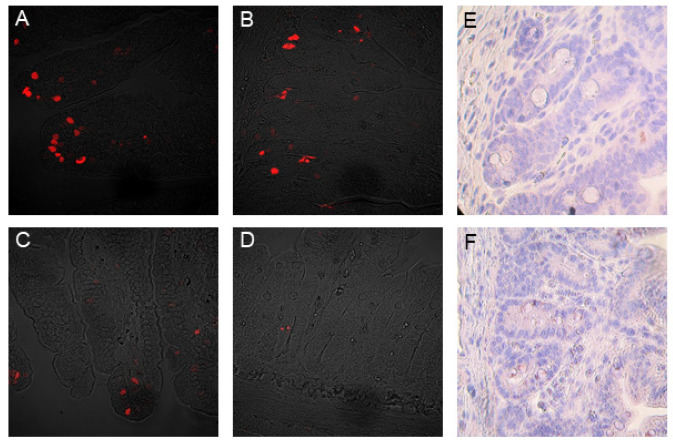
Representative microscopic images of the middle jejunum mucosa in 10-day-old control (**C**) and sodium-butyrate-treated piglets (**B**) (at a dose of 0.3%), with apoptotic cells (red) visualized using a TUNEL assay. Intestinal villi (**A**) and crypts (**B**) in piglets from the control group (group C). Intestinal villi (**C**) and crypts (**D**) in piglets from the sodium butyrate treated group (group B). The tissue structure was visualized using Nomarski contrast in a confocal microscope (LSM Pascal, Zeiss, Germany, magnification: 400×). Ki67-positive epithelial cells (3,3′-diaminobenzidine staining, brown) in crypts counterstained with hematoxylin in blue in animals from the control group (group C) (**E**) and from the sodium-butyrate-treated group (group B) (**F**). Light microscope (Axioskop 40, Zeiss, Germany), magnification: 400×.

The mitotic and apoptotic indices are presented in Table 3. In the duodenum, no significant differences in the mitotic and apoptotic indices of crypts and villi were observed between experimental groups. In contrast, in the middle jejunum, supplementation of milk formula with sodium butyrate significantly increased the crypt mitotic index and the villi apoptotic index compared to the control group. The representative images of the effect of sodium butyrate milk supplementation on the induction of physiological cell death and the proliferation of crypt stem cells in the small intestine mucosa are shown in Figure 3.

### 3.6. Muscarinic ACh Receptors

**Figure 4:**
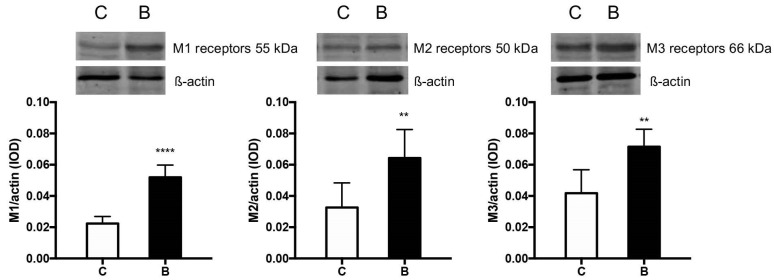
Quantitative results of optical density and representative Western blots of M1, M2, and M3 receptor expression in the lysates from the mucosa of the middle jejunum segments in newborn pigs. Piglets fed milk formula alone (group C) or supplemented with sodium butyrate at a dose of 0.3% (group B). Values are given as means ± SEM; * indicates statistical differences between groups when ** *p* < 0.01, **** *p* < 0.0001.

Milk formula supplemented with sodium butyrate significantly increased the cytoplasmic expression of all types of receptors (M1, M2, and M3) in the homogenates from the middle jejunum of newborn piglets (Figure 4).

## 4. Discussion

The intestine plays a key role in neonatal growth and health, including metabolic programming. In the present study, we showed that sodium butyrate stimulates small intestinal contractility in neonatal piglets fed milk formula lacking butyrate. To our knowledge, ours is the first study to demonstrate duodenal and jejunum contractility in newborn piglets in response to oral administration of sodium butyrate.

It has previously been reported that milk formula feeding slows down intestinal maturation, including the development of intestinal motility patterns, in newborns, leading to reduced number of noradrenergic fibers in the enteric nervous system [24], decreased responsiveness to ACh-stimulated intestinal contractility [25], and lower percentage of normal slow waves within the small intestine [26]. Oral treatment with butyrate had a similar effect on the contractility of both the intestinal segments assessed in the current study. A significant increase in the amplitude of both spontaneous and ACh-stimulated contraction was observed in both the duodenum and middle jejunum after butyrate treatment. Similarly, in both intestinal segments, EFS impulses resulted in significantly increased amplitude of contractions. This suggests enhanced motor activity, which may consequently affect the mixing and propulsion of luminal contents. This, in turn, may result in increased nutrient digestibility and improved growth performance. Indeed, final BW and daily BW gain were significantly higher in piglets receiving sodium butyrate compared to control piglets, which is in line with results from previous experiments in neonatal [20,27] and weaned piglets [28]. A previous study by Manzanilla et al. [29] suggested that jejunum motility is not directly affected by the oral administration of butyrate as it is quickly absorbed by the epithelial cells of the stomach and upper small intestine; thus, it is doubtful that significant amounts of butyrate reach the middle part of the jejunum [29]. The reported effects of orally administered sodium butyrate on both the structure and function of the small intestine may possibly be explained by the increased flow of butyrate into the duodenum, as previously observed in studies on sheep [30].

The mechanism of butyrate’s effect on intestinal contractility at a cellular and molecular level is poorly understood. Most of the data available on butyrate are related to its effect on ileal and large bowel muscular activity, with both excitatory [13,14,15,18,31,32] and inhibitory [16,17] effects having been reported. Contradicting results may depend on the different experimental approaches, models, and doses of butyrate used [10,32].

There are indications that butyrate may act directly on smooth muscle cells [14,16,33] as well as indirectly via extrinsic or intrinsic afferent nerves and myenteric neurons [18,19,31,34]. It should be mentioned that, in a few previous studies, removal of the mucosa from intestinal preparations inhibited butyrate-induced contractility, suggesting the presence of sensory mechanisms in the epithelium [13,31].

Thus, we decided to study full–thickness intestinal segments instead of only muscle preparations as previous in vitro studies have revealed that different types of ion channels and receptors, as well as interneurons and nonmuscle cells, occur along the intestinal wall and may be crucial for the contractile response [35]. Moreover, receptors sensitive to SCFA (FFAR2 and FFAR3) have been identified in several cells within the intestinal wall, including enteroendocrine cells, enteric neurons, mast cells, and leukocytes [32,36].

Many studies have shown that butyrate enhances enteric cholinergic and noncholinergic neurons [13,18,31], which may be mediated by changes in the levels of histone acetylation or the gene expression of neuromediators [13]. We investigated the expression of muscarinic receptors belonging to two classes that differed in terms of their response to ligand binding. Briefly, activation of odd-numbered MRs (M_1_R, M_3_R, and M_5_R) stimulates phospholipid turnover and increases intracellular calcium levels, while activation of even-numbered MRs (M_2_R and M_4_R) inhibits adenylyl cyclase activity, thereby reducing levels of intracellular cAMP. Previous studies have shown that butyrate stimulates GH (by increasing Ca^2+^ and reducing GHRH-stimulated cAMP accumulation). Moreover, GH secretion is modulated by cholinergic pathways and is responsive to pharmacological suppression by muscarinic receptor blockade [37]. We demonstrated that butyrate supplementation increased the expression of both types of muscarinic receptors (M1 and M2) in the middle jejunum. This shows how butyrate may act as an important integrator of cellular signaling in the intestine. On the other hand, the significantly higher amplitude of contraction observed in the butyrate-treated piglets in the present study after pretreatment with atropine and TTX also indicates the involvement of a noncholinergic pathway.

It should be mentioned that, in our study, the contractility characteristics poorly correlated with changes in the intestinal muscle layer. We reported an increase in the thickness of the muscularis mucosa in the duodenum, with the lack of a similar trophic effect in the middle jejunum. Therefore, the effect of sodium butyrate on duodenal motility could be related to its direct trophic impact on the intestinal smooth muscle cells. A similar effect was observed in the muscularis layer of the duodenum in piglets receiving butyrate [20], but these were not pigs assessed shortly after weaning as in the present study [28]. A complementary explanation for the action of butyrate might be one of an increased level of intestinal hormones. Butyrate treatment in neonatal piglets has been shown to elevate plasma cholecystokinin levels [20] as well as glucagon-like peptide-2 concentrations [21], and their trophic effect on the small intestine has been demonstrated in newborn piglets and calves [21,38].

However, many studies on butyrate have demonstrated an increase in the mass of the distal jejunum, ileum, and large intestine, including thickening of the mucosa and muscle hypertrophy [16,20]. This trophic effect of butyrate may also be indirectly mediated through a neurohormonal mechanism [34]. Moreover, Suply et al. [18] noted that, in the early postnatal period, there is an aboral gradient in the maturation of myenteric neurons between the proximal and distal colon, which may also be true for the region-specific sensitivity to butyrate in the current study. It should also be noted that the results obtained from in vitro experiments may differ from those from in vivo experiments because, for example, duodenal motility is highly dependent on vagal innervation and gastric motility. Interestingly, plasma concentration of pancreatic polypeptide, a marker of vagal efferent activity, increased more than 2-fold in piglets receiving sodium butyrate [20].

In the middle jejunum, butyrate also increased intestinal apoptosis as well as the proliferation of crypt stem cells, which is in part consistent with the data from Guilloteau et al. [39] in young calves but contrary to those of Le Gall et al. [28], who noticed increased crypt stem cell proliferation in weaned pigs. This may be due to the different age of animals and different dynamics of small intestinal epithelium renewal. In the early postnatal period, increased proliferation and apoptosis facilitates the process of the replacement of fetal enterocytes with adult enterocytes and the maturation of the epithelium barrier. These data may imply accelerated intestinal mucosa growth and maturation or repair after injury. Increases in villi height and crypt depth were previously reported in studies on piglets treated with sodium butyrate [20,21,40]. Increased proliferation index might also be associated with butyrate function as an energy source for intestinal cells or its stimulating effect on GI peptides and growth factors [10].

## 5. Conclusions

The current study provides evidence that dietary sodium butyrate has a wide spectrum of effects in the gastrointestinal tract, including increasing small intestinal contractility. Our findings indirectly provide data showing that breast milk sodium butyrate may stimulate gut motility in human infants. Supplementation of milk formula with sodium butyrate may reverse the negative effects of milk formula in the upper intestine, including the reduced motility usually associated with milk formula feeding. Immature intestinal motor patterns and enteric nervous system function have been reported in very low birth weight and preterm infants, and GI motility disorders are a common feature of infancy [41]. Abnormal motility patterns, either due to enteric neuropathy or myopathy, are observed in Hirschsprung’s disease, chronic intestinal pseudo-obstruction, slow transit constipation, or Crohn’s disease [41,42]. The direct trophic effect of butyrate in the duodenum may be an additional field of interest for future research. Further research is also needed to determine the potential use of butyrate during the developmental period in clinical medical practice, for example, in the treatment of intestinal motility disorders in newborns. Modification of milk formulas to promote the development of proper motility patterns in the GI tract is of great practical importance.

## Figures and Tables

**Table 1:** Electrical field stimulation (EFS) induced amplitude of contraction (mm) in the presence or absence of tetrodotoxin (TTX (10^−6^ M)) in duodenal and middle jejunum fragments in control (C) and sodium-butyrate-treated piglets (B). Values are given as means ± SD; * indicates statistical differences between groups when * *p* < 0.05, ** *p* < 0.01, *** *p* < 0.001; ^§^ indicates statistical differences between groups in the presence of TTX when ^§^ *p* < 0.05, ^§§^ *p* < 0.01, ^§§§^ *p* < 0.001.

	Amplitude of EFS-Induced Contraction (mm)					
	No Additional Treatment			TTX		
	0.5 Hz	5 Hz	50 Hz	0.5 Hz	5 Hz	50 Hz
Duodenum						
C	1.67 ± 0.57	1.89 ± 0.76	2.08 ± 0.58	0.79 ± 0.32 ^§§^	0.84 ± 0.34 ^§§^	1.38 ± 0.25 ^§^
B	2.90 ± 0.50 ***	3.30 ± 0.59 ***	3.53 ± 0.74 ***	1.15 ± 0.17 *^§§§^	1.55 ± 0.23 ***^§§§^	1.73 ± 0.24 *^§§§^
*p* value	0.0004	0.0009	0.0007	0.014	0.0002	0.014
Middle jejunum						
C	1.23 ± 0.24	1.73 ± 0.66	1.91 ± 0.36	0.59 ± 0.10 ^§§§^	0.61 ± 0.14 ^§§§^	1.11 ± 0.20 ^§§§^
B	1.91 ± 0.36 ***	2.34 ± 0.26 *	2.79 ± 0.54 *	0.74 ± 0.08 **^§§§^	0.96 ± 0.19 ***^§§§^	1.38 ± 0.25 *^§§§^
*p* value	0.0006	0.030	0.011	0.003	0.001	0.030

**Table 2:** Amplitude of contractions (mm) of acetylcholine (ACh)-stimulated or atropine-treated duodenal and middle jejunum fragments in control (C) and sodium-butyrate-treated piglets (B) at a dose of 0.3% on day 10 of life. Values are given as means ± SD; * indicates statistical differences between groups when * *p* < 0.05, ** *p* < 0.01, *** *p* < 0.001.

	ACh^−9^	ACh^−8^	ACh^−7^	ACh^−6^	ACh^−5^	ACh^−4^	ATR^−6^
Duodenum							
C	4.5 ± 0.5	4.5 ± 0.5	4.6 ± 0.5	4.8 ± 0.5	6.1 ± 1.2	8.2 ± 1.0	0.9 ± 0.2
B	5.4 ± 1.1 *	5.6 ± 0.9 **	6.1 ± 0.9 ***	6.2 ± 1.0 ***	7.4 ± 1.4 *	11.0 ± 1.9 ***	2.2 ± 0.4 **
*p* value	0.022	0.002	0.0001	0.0005	0.025	0.0004	0.001
Middle jejunum							
C	3.3 ± 0.8	3.4 ± 0.9	3.4 ± 1.0	3.6 ± 0.9	4.8 ± 1.6	8.9 ± 2.4	0.6 ± 0.1
B	5.3 ± 1.5 ***	5.4 ± 1.5 ***	5.9 ± 1.5 ***	6.4 ± 2.1 ***	7.6 ± 2.3 **	11.9 ± 3.4 *	2.0 ± 1.2
*p* value	0.0004	0.0009	0.0001	0.0002	0.003	0.034	0.342

**Table 3:** The mitotic and apoptotic indices in control (C) and sodium-butyrate-treated piglets (B). Values are given as means ± SD; Indicates statistical differences between groups when ** *p* < 0.01; ^§^ indicates statistical differences between intestinal segments when ^§^ *p* < 0.05.

Group/Segment	Duodenum	Middle Jejunum	p Value
Mitotic index			
C	1.11 ± 0.48	0.88 ± 0.35	0.181
B	1.37 ± 0.26	1.32 ± 0.20 **	0.622
*p* value	0.271	0.001	
Apoptotic index—crypts			
C	3.19 ± 0.45	3.11 ± 0.69	0.722
B	2.87 ± 0.61	3.43 ± 0.93	0.105
*p* value	0.153	0.358	
Apoptotic index—villi			
C	3.42 ± 0.41	3.28 ± 0.58	0.487
B	3.13 ± 0.57	2.51 ± 0.51 **^§^	0.011
*p* value	0.165	0.002	

## Supplementary Materials

The following supporting information can be downloaded at: https://www.mdpi.com/article/10.3390/nu14204301/s1. Figure S1: The scheme of an in vitro study on isolated fragments of the small intestine of newborn piglets; Figure S2: Electric field stimulation (EFS) induction of contraction amplitude (mm) in the duodenum and middle section of the jejunum of piglets.
