# Supplementary material for: Milk Formula Enriched with Sodium Butyrate Influences Small Intestine Contractility in Neonatal Pigs
Source: Nutrients. 2022 Oct 14;14(20):4301. doi: 10.3390/nu14204301 (PMC9608939; doi:10.3390/nu14204301)
Supplement: Supplementary file 1 [file nutrients-14-04301-s001.zip › nutrients-1943778-supplementary.pdf]

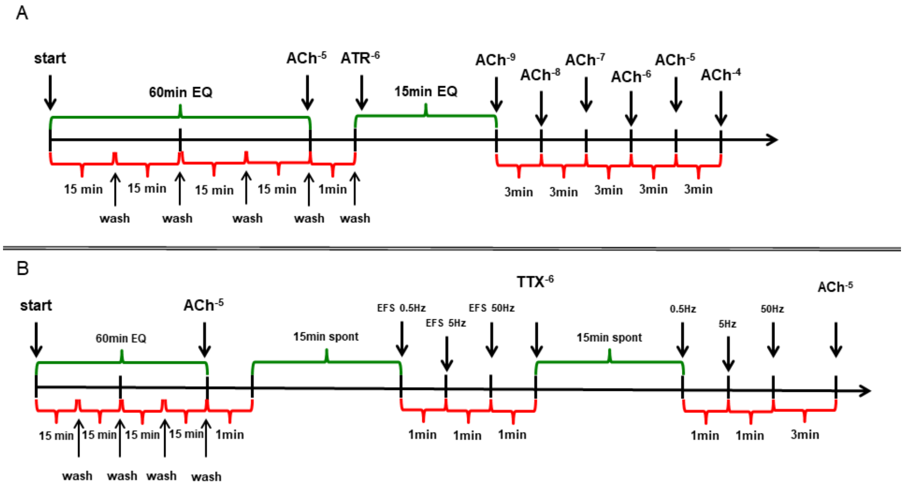

Figure S1. The scheme of an in vitro study on isolated fragments of the small intestine of newborn piglets.

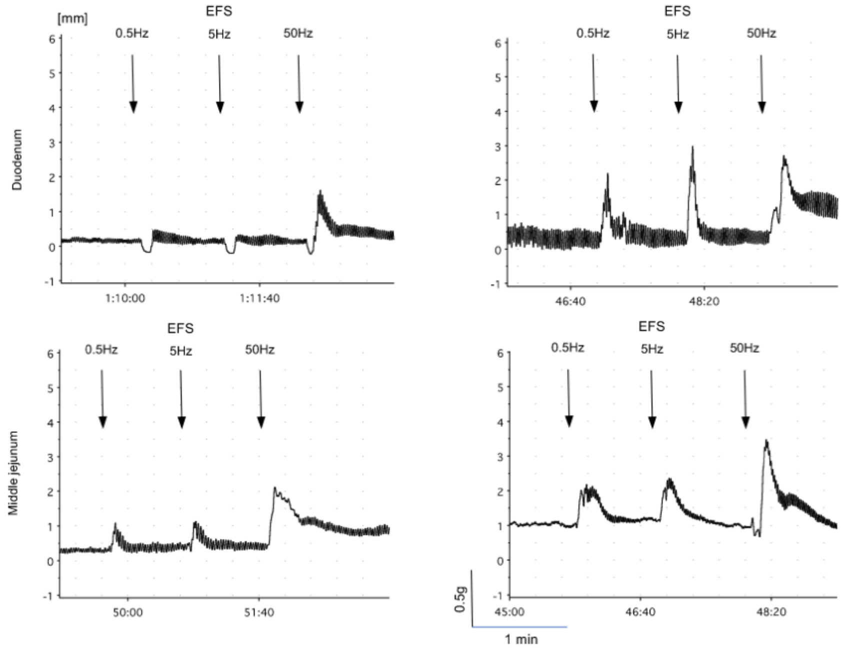

Figure S2. Electric field stimulation (EFS) induction of contraction amplitude (mm) in the duodenum and middle section of the jejunum of piglets.
